# Hemiplegia Following Fluid Administration Through an Implanted Venous Access Device: A Case Report

**DOI:** 10.5811/cpcem.2021.12.55230

**Published:** 2022-01-28

**Authors:** James Waymack, Christopher McDowell, Nida Feller, Sharon Kim

**Affiliations:** * Southern Illinois University School of Medicine, Department of Emergency Medicine, Springfield, Illinois; † Southern Illinois University School of Medicine, Center for Clinical Research, Springfield, Illinois

**Keywords:** hemiplegia, central venous catheter malposition, case report

## Abstract

**Introduction:**

Many patients seen in the emergency department (ED) have central venous access placed or previously established placement. Catheters inadvertently placed in the arterial circulation may lead to complications or adverse events.

**Case Report:**

We present a case of hemiplegia in a 63-year-old man following intravenous fluid administration through a malpositioned catheter that was initially unrecognized. The patient initially presented to the ED for stroke-like symptoms and was discharged following workup. On a subsequent visit for similar symptoms, intra-arterial placement of the catheter was diagnosed.

**Conclusion:**

It is important for emergency physicians to be aware of this potential complication of central venous cannulation and that arterial malposition of a previously placed central line may go unrecognized with the potential to cause cerebral ischemia when cerebral blood flow is reduced by the infusion of intravenous fluids or medications.

## INTRODUCTION

Central venous lines are commonly placed in emergency department (ED) patients, and many patients seen in the ED with chronic conditions have indwelling central venous devices. We present a case of hemiplegia following intravenous (IV) fluid administration through an implanted venous device that had been inadvertently placed in the subclavian artery and unrecognized as such until malposition was identified on computed tomography angiography (CTA) of the chest following a stroke evaluation. Awareness of this potential complication and how it may present as transient neurologic symptoms is important to the emergency physician who routinely places central venous lines.

## CASE REPORT

A 63-year-old male with a history of stage IV metastatic esophageal adenocarcinoma and recent placement of an implanted venous access device three weeks prior presented to the ED from the infusion clinic at the hospital with a chief complaint of left-sided arm and leg weakness. The patient had no known cerebral involvement of metastatic disease. The patient, who was receiving IV fluids for rehydration, noted left-sided weakness upon attempting to rise to use the restroom. He was emergently transported to the ED for evaluation. Upon arrival he confirmed the left-sided weakness without other motor deficit reported. He denied visual disturbance or headache. He denied numbness or tingling of the face or extremities.

The patient noted that he had been seen in the ED two weeks prior for a similar presentation of left-sided weakness with urinary incontinence and possible left leg shaking while receiving chemotherapy via the same port. During the prior evaluation a stroke team was activated with emergent neurologic consultation. Non-contrast computed tomography (CT) of the head was negative, but the patient was unable to undergo magnetic resonance imaging (MRI) of the brain due to extreme gastric reflux as a complication from previous surgery for his esophageal cancer. No head or neck CTAs were obtained. He was observed overnight, the left-sided weakness was resolved, and he was subsequently diagnosed with Todd’s paralysis. Levetiracetam was prescribed, and he had been compliant in taking the medication since the previous visit.

Upon arrival at the second visit his vital signs included a temperature of 36.5° degrees Celsius, blood pressure of 131/78 millimeters of mercury, heart rate of 82 beats per minute, respirations at 18 breaths per minute, and an oxygen saturation of 97% on room air. His blood glucose level was 96 milligrams per deciliter (mg/dL) (reference range: 70–105 mg/dL). Physical examination was significant for no movement of the left arm and no effort against gravity of the left leg, giving him a National Institutes of Health Stroke Scale score of 7. The remainder of the neurologic exam including level of consciousness, orientation, speech, visual exam, and right-sided strength and coordination were normal. A stat stroke activation was declared with neurology consultation.

**Image 1:**
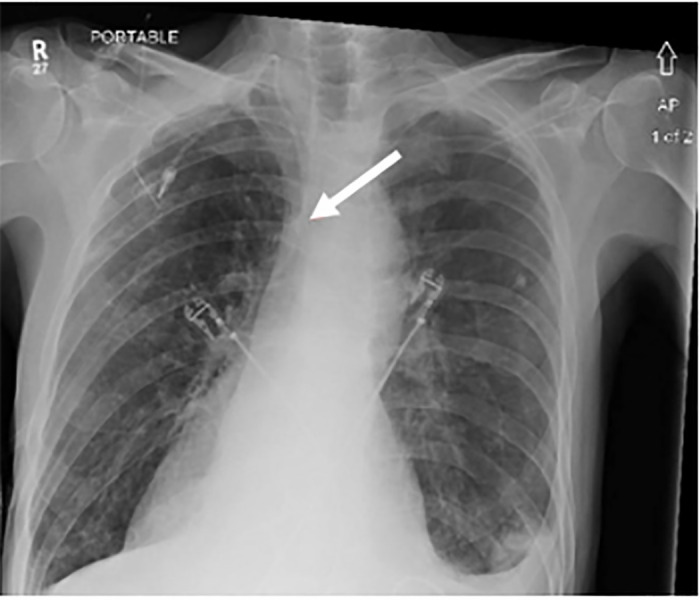
A chest radiograph depicting the right-sided chest port (arrow) with line coursing medially.

A non-contrast CT of the head was obtained with no acute abnormality. Plain chest radiograph demonstrated a right-sided port with central line coursing medially, suggesting a possible arterial course (Image 1). The patient again was unable to tolerate MRI of the brain. Due to recurrence of his symptoms and the situational similarities (both episodes occurring during infusions through his port) CTA imaging of the chest, neck, and head were obtained to evaluate for vessel stenosis or catheter malposition.

**CPC-EM Capsule**

What do we already know about this clinical entity?

*Central venous access catheters or devices are commonly used in medicine. Complications from malposition of these devices may lead to adverse events*.

What makes this presentation of disease reportable?

*We present a case of an unrecognized, arterially placed central catheter that presented as transient hemiplegia following the administration of intravenous fluid through the catheter*.

What is the major learning point?

*Chest radiography may not obviously identify an arterially placed central venous catheter. Subsequent Computed tomography angiography may be helpful*.

How might this improve emergency medicine practice?

*This case highlights the need to confirm appropriate catheter placement and consider implanted devices as a contributing source of a patient’s presentation*.

**Image 2:**
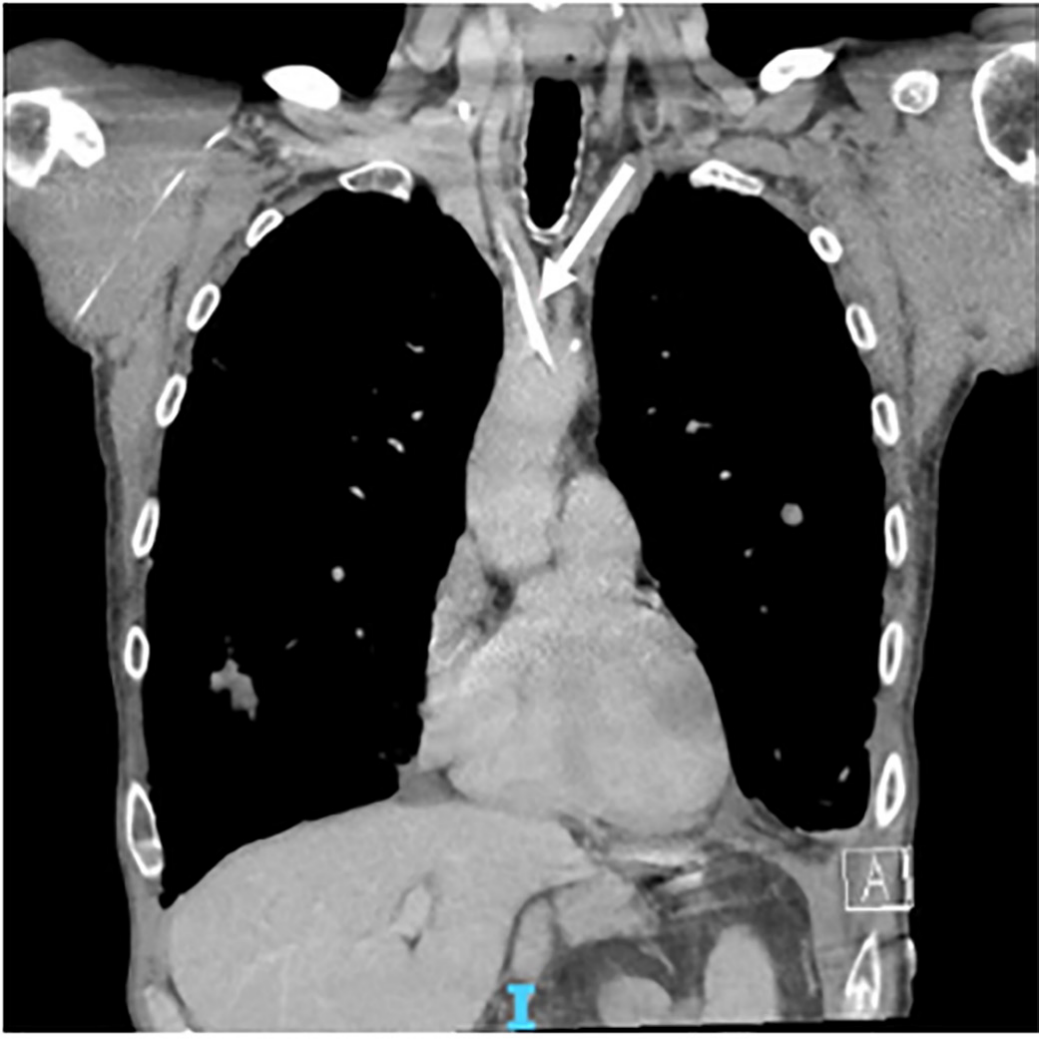
Coronal view of a computed tomography angiogram depicting the central venous line coursing through the internal carotid artery and terminating at the aortic arch (arrow).

**Image 3:**
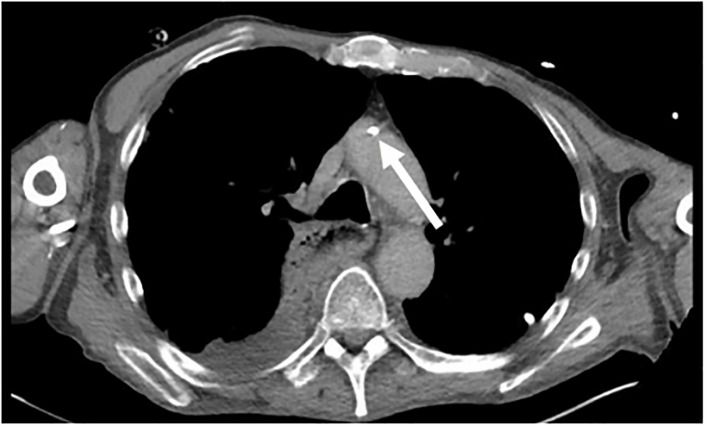
Axial view of a computed tomography angiogram depicting the central venous line tip seen in the aortic arch (arrow).

The CTA revealed a right-sided chest port in place appearing to enter the right subclavian artery with the tip located in the anterior aspect of the aortic arch (Images 2 and 3). Vascular surgery was consulted to see the patient. During the patient’s stay in the ED his symptoms improved within three hours to normal strength on the left side. He subsequently underwent operative removal of the malpositioned catheter from where it was found to have initially entered the internal jugular vein, went through the back wall and through a smaller possibly vertebral vein. The catheter entered arterial circulation through a vertebral artery and then traveled into the right subclavian artery. The port and catheter were removed entirely, vascular defects were repaired, and the patient recovered from the procedure well with no circulatory or neurologic deficit. He was diagnosed with transient ischemic attack due to infusion through an intra-arterial catheter and was discharged home on hospital day five.

## DISCUSSION

We report a case of a patient with transient cerebral ischemia presenting as hemiplegia following infusion of IV fluids through a subcutaneous venous access device that had been inadvertently placed arterially and initially unrecognized. It is suspected that intra-arterial infusion led to hemodilution and decreased cerebral oxygen delivery, resulting in the patient’s presentation with neurologic deficits.^1^ While accidental arterial puncture or cannulation is often cited and common knowledge, a review of the literature did not reveal any cases similar to ours.

Cannulation of the central veins with subsequent placement of a catheter is commonplace in medicine. More than five million centrally accessed venous lines are placed each year with approximately 8% of hospitalized patients requiring the procedure.^2^,^3^ Complication rates for central venous access are around 15% to include pneumothorax, arterial puncture or cannulation, air embolism, arrhythmia, infections, thrombosis, or medical device embolization.^4^ Arterial injury occurs in 3–12% of central venous catheter placements.^5^–^8^

Subcutaneous ports or implanted venous access devices are often placed in the anterior chest wall with an internal jugular or subclavian catheter. These devices are chosen for patients who will need long-term central venous access. Venous access is obtained in the usual fashion with the Seldinger technique for catheter placement; however, a subcutaneous pocket is made for the port device to be implanted and attached to the catheter. This procedure is often performed by a surgeon or interventional radiologist in a non-emergent setting. Complications from port placement occur more often due to infection than arterial injury or malposition. One single-center study of 117 insertions reported infection as the primary complication and reason for premature port removal.^9^

The operative report for port placement stated dark pulsatile blood was encountered on initial puncture; therefore, ultrasound was used to demonstrate that the wire was seen in the internal jugular vein. A venogram was then performed showing the catheter in the right internal jugular vein with an abnormal-appearing superior vena cava. It was concluded that these findings were due to venous hypertension, and the port insertion was completed. Initial postoperative images were interpreted as the line terminating at the brachiocephalic junction. On further retrospective chart review of our case, radiography demonstrates the catheter coursing toward the midline, which could be concerning for arterial cannulation and catheter malposition.

Appropriate venous cannulation is often localized or guided by ultrasound and initially visually confirmed by return of deoxygenated-appearing blood. However, clinical judgment may only have an accuracy of 70% for malposition.^10^ Chest radiography is commonly used to rule out pneumothorax and to confirm appropriate line placement by noting the course of the line through the approaching vasculature with termination in the superior vena cava or cavoatrial junction at the right heart border. Some studies have questioned the quality and accuracy of chest radiography, primarily focusing on its utility for identification of pneumothorax.^11^,^12^ Bailey et al found in 184 central venous catheter placements a complication rate of 9%, most commonly malposition, and that clinician gestalt and fewer than three needle passes correlated with an absence of complication.^11^ Those placing central venous lines may need to consider multiple modalities to confirm appropriate venous cannulation depending on comfort and potential complicating factors of each line placement.

We share this case as many patients have central venous access placed, and EDs encounter many patients with neurologic deficits that may be due to various underlying causes. The root cause of this patient’s ischemia was not identified on his first presentation of transient ischemia, further evidence that this constellation of procedural complications and subsequent symptomatology was not clearly evident to those involved in his care.

## CONCLUSION

This case of a patient presenting with transient neurologic symptoms due to intravenous fluid administration through an intra-arterial central venous line highlights the importance of considering uncommon causes of a patient’s presentation. It is important for emergency physicians to be aware of this potential complication of central venous cannulation and that arterial malposition of a previously placed central line may go unrecognized, with the potential to cause cerebral ischemia when cerebral blood flow is reduced by the infusion of IV fluids or medications.
